# Mobile Heat Early Warning System for Pregnant and Postpartum Women in South Africa: Prospective Observational Evaluation Study

**DOI:** 10.2196/102863

**Published:** 2026-09-25

**Authors:** Clara Sofia Heil, Shobna Sawry, Susanne Frennert, Jean Le Roux, Pascalia Ozida Munyewende, Sibusiso Mkwananzi, Ijeoma Solarin, Perkins Watambwa, Koen van der Sanden, Isabelle Lange, Günter Alce, Veronique Filippi, Stanley Luchters, Chuansi Gao

**Affiliations:** 1 Aerosol and Climate Laboratory, Division of Ergonomics and Aerosol Technology, Department of Design Sciences Faculty of Engineering Lund University Lund, Skåne Sweden; 2 Climate and Health, Implementation Science Faculty of Health Sciences University of the Witwatersrand Johannesburg, Gauteng South Africa; 3 Centre for Sexual Health and HIV AIDS Research Harare, Harare Zimbabwe; 4 Netherlands Organisation for Applied Scientific Research Soesterberg, South Holland The Netherlands; 5 London School of Hygiene & Tropical Medicine London, England United Kingdom; 6 Department of Neurology Technical University of Munich Munich, Bavaria Germany; 7 Department of Public Health and Primary Care Ghent University Ghent, Flanders Belgium; 8 Liverpool School of Tropical Medicine Liverpool, England United Kingdom

**Keywords:** pregnancy, postpartum period, hot temperature, mobile apps, digital health, maternal health, global warming, South Africa

## Abstract

**Background:**

Early warning systems (EWS) have the potential to reduce heat-related health risks, yet few are designed specifically for vulnerable populations. Pregnant women and infants, often cared for by postpartum women, are particularly susceptible to heat-related illnesses. The MotherHeat Alert mobile app is a heat EWS specifically developed for pregnant and postpartum women.

**Objective:**

This study aimed to assess the usability and effectiveness of the MotherHeat Alert in the Tshwane District, South Africa.

**Methods:**

This prospective observational evaluation study included a cohort of 201 participants (100 pregnant and 101 postpartum) who were followed over a 6-month period. In addition, a questionnaire was administered at 3 time points (baseline, midpoint, and end point) to collect self-reported information on health, living situation, and heat exposure, among other parameters. Usability was evaluated in the end point questionnaire through a validated Post-Study System Usability Questionnaire (PSSUQ), using descriptive statistics. Effectiveness was assessed through changes in self-reported heat-protective behaviors between baseline, midpoint, and end point, as well as through end point assessments of heat-health knowledge increase and self-reported behavior change attributable to the intervention. Generalized estimating equations were used to analyze repeated measures of heat-protective behaviors, while descriptive statistics were used to summarize end point–reported knowledge gains and behavior change.

**Results:**

Usability scores indicated high overall usability, with mean PSSUQ scores of 1.76 (SD 0.60) among pregnant women and 1.37 (SD 0.45) among postpartum women. Similar patterns were observed across most PSSUQ subscales, including system usefulness, interface quality, and information quality. Most self-reported behavior change due to alerts received through the app was rated as large to very large by the pregnant women (47/70, 67%) and postpartum women (68/82, 83%). Nevertheless, comparing behavior change at baseline and end point mainly highlighted a decrease in protective behavior in both pregnant and postpartum cohorts. Self-reported knowledge increase among pregnant and postpartum women was reported as large to very large following the use of the EWS (55/70, 79% and 72/82, 88%, respectively). Importantly, however, attrition was substantial, particularly among pregnant participants, reaching 30% (30/100) in the pregnant cohort and 19% (19/101) in the postpartum cohort by end point.

**Conclusions:**

The MotherHeat Alert app demonstrated positive outcomes in usability. Effectiveness showed conflicting results, with positive self-reports on behavior change and knowledge increase, yet a decrease in protective heat behaviors when comparing baseline and midpoint with end point results, likely due to seasonality effects. Nevertheless, these findings highlight the potential of tailored EWS to support heat adaptation among pregnant and postpartum populations. Successful implementation and scalability require careful consideration of contextual factors, further assessment of long-term usability and integration into existing health infrastructure.

## Introduction

Heat exposure causes an estimated 546,000 deaths annually worldwide [1], and contributes to increases in morbidity, particularly among high-risk populations [2] such as pregnant women. With heat extremes becoming longer, more frequent, and more intense, heat extremes currently classified as rare will become increasingly commonplace [3,4]. Pregnant women face heightened physiological strain due to elevated core body temperature and metabolic demands, while postpartum women bear responsibility for protecting heat-vulnerable newborns who cannot thermoregulate effectively. Many women in Africa have limited or no protection against heat during pregnancy [5], outlining the urgent need to identify effective prevention and response measures in these regions [3]. Heat action plans and forecasts, including heat-related early warning systems (EWS), issue early alerts and advisories [6,7] and have been demonstrated to promote heat adaptation and save lives [7-10]. However, current EWS designed to reduce heat-related health risks remain largely generic [9,10]. Despite heat-health risks in pregnancy, no heat-health EWS has been systematically designed and evaluated for pregnancy and the perinatal period.

EWS are widely recognized as cost-effective tools for climate adaptation, integrating different aspects of risk knowledge, monitoring and warning, and communication and response capacity [8]. A central challenge for EWS effectiveness is risk communication; systems must ensure that people at risk receive, comprehend, and respond to the alerts [11] communicated at the community and individual level [12]. For EWS to function effectively across different geographies and cultures, they should integrate social and cultural perceptions shaping heat awareness, risk perception, protective behaviors, and coping strategies, while providing science-based and locally relevant warnings [8]. Additionally, warnings issued without advice on responses are less effective [13]. Evidence from sub-Saharan Africa suggests that digital health interventions are most successful when they are simple, low-cost, and aligned with local contexts and languages [8,14,15]. Successful implementation further depends on user attitudes and behaviors and cultural acceptance [16]. Interventions must therefore be people-centered, context-sensitive, and coproduced with communities and local partners to ensure warnings are understood and acted upon [8,14,15]. Integrating such systems into existing digital health strategies may further support adoption and sustained engagement [14].

While there seems to be some awareness regarding heat warnings and high temperature forecasts among the general population, taking protective actions is less common [17], particularly among individuals who do not see themselves as at risk [13]. There is a demand for assessing the effectiveness of interventions to determine their ability to change knowledge, awareness, and behavior. Limited awareness of heat-induced health risks poses a major challenge for heat EWS [10,18]. Increasing the understanding of these risks and adopting small behavior changes could significantly reduce heat-induced morbidity and mortality [18]. Pregnant women exposed to cues to action are more likely to exhibit positive adaptation behaviors in response to heat, influenced by the awareness of vulnerabilities and their risk perception [18]. Therefore, effective communication of information enhances awareness while also significantly leading to adoption of adaptive behaviors [18]. Furthermore, research stresses the importance of assessing the effectiveness of heat EWSs, not just overall, but of specific interventions, particularly targeting vulnerable populations [10]. Improving the design and implementation of EWS directly enhances their effectiveness [10] and usability, which refers to user satisfaction within a defined intended use and context of use [19].

This study evaluates the usability and effectiveness of a mobile heat EWS app implemented in a South African context, with messages co-designed with pregnant and postpartum women. It is part of the larger Heat Indicators of Global Health (HIGH) Horizon project [20], which developed this EWS to help protect pregnant and postpartum women from heat exposure [21]. We assess whether the warning system is perceived as useful by its intended users, whether the mHealth app can improve awareness of the adverse health consequences of heat during and shortly after pregnancy, and whether it supports behavior change toward more protective heat-health practices.

## Methods

### Study Design and Setting

We conducted an intervention cohort study to evaluate the usability and effectiveness of a personalized heat-health EWS, MotherHeat Alert, among 100 pregnant and 101 postpartum women in South Africa. As part of the larger HIGH Horizons project [20], the study was implemented between October 2024 and August 2025 at a community health center (CHC) in Tshwane, Gauteng Province, South Africa. The district is predominantly urban, with large informal settlements. Housing consists of formal houses, backyard rooms, and informal dwellings, most of which lack consistent access to cooling infrastructure. The telecom industry reports that almost 98% of mobile connections in South Africa are connected via 3G, 4G, or 5G mobile networks [22]. Average daily outdoor temperature was estimated using Copernicus Climate Data for coordinates near the health facility during the peak summer period (December 2024 to January 2025), when temperatures are typically highest in the study setting. During this period, average monthly maximum temperatures ranged from 26.9°C to 27.4°C, with 19 days measuring above 30°C and 3 days above 35°C.

### Study Participants

A total of 2 cohorts were enrolled in this study:

1. Pregnant women: Confirmed pregnant, in the second trimester of pregnancy as assessed by a registered health worker, aged 18 years and older, and attending antenatal care services at the CHC.
2. Postpartum women: Women aged 18 years and older, with a live-birth infant in the past 3 months or women with a child aged 6-9 months of age, and recruited from the postnatal care services or well-baby and immunization clinics at the CHC.

Additionally, all participants needed to own a smartphone that could run the MotherHeat Alert app, intend to remain in the study area for the next 6 months, and be able and willing to provide written informed consent for study participation.

### Sampling and Recruitment

Convenience sampling was used to recruit participants, with efforts made to cover diversity in age and demographic background. Recruitment flyers and posters were displayed in the maternity unit and health facilities, containing brief details about the study and contact details. Languages spoken throughout recruitment were English, Ndebele, Sepedi, Setswana, Zulu, and Sesotho.

### Study Visits

Questionnaire data were collected at 3 study visits (baseline, 2 months, and 6 months). The enrollment visit (~60 to 90 minutes) included informed consent, administration of the baseline questionnaire, and a 30-minute training session to install and configure the MotherHeat Alert app and show participants how to use the app. User profiles were set up within the app to include age, body weight, and height. Follow-up visits occurred at 2 and 6 months post enrollment, January-March and June-August, respectively. Slight changes were made between trial registration and the implementation of the study by introducing visit windows to reduce the risk of missed visits and participant dropouts. Each of these sessions lasted approximately 60 minutes and included the questionnaire administration. At the 6-month visit, participants also completed the Post-Study System Usability Questionnaire (PSSUQ) to assess the usability of the EWS (listed in Multimedia Appendix 1). At any time during the study period, participants could complete a short in-app survey, consisting of 7 questions. This survey was optional and assessed heat symptoms, perceived heat, and app usability, collecting real-time submission of user responses.

### Data Collection and Analyses

Quantitative data were collected through structured, interviewer-administered questionnaires using Research Electronic Data Capture (REDCap; Vanderbilt University) [23] and through the continuous in-app data collection. Questionnaires captured sociodemographic information, medical and obstetric history, knowledge and protective behaviors linked to heat risks, data on the economic burden and app feasibility, and acceptability and usability, particularly at the end point visits. The in-app data included timestamps of interactions, app usage metrics, and responses to usability and heat-related questions. For this study, usability, knowledge increase, and behavior change related to the intervention were of main interest. Furthermore, the EWS captured heat parameters, which were used to analyze 2 in-app survey items, as described in more detail in the following section. The current Universal Thermal Climate Index (UTCI), a comprehensive heat stress index integrating air temperature, humidity, solar radiation, and wind speed and including models estimating clothing insulation and metabolic heat production [24], was used to capture the environmental heat exposure in this study. The app retrieved local weather forecast data daily at approximately 6 AM, but an alert was only generated and issued when the predefined heat threshold was met. Alert generation was determined locally on participants’ devices.

### Outcome Measures: Usability and Effectiveness

Usability was assessed through a validated PSSUQ administered at the end point visit. The PSSUQ is a 16-item scale that assesses user satisfaction through an overall evaluation of the system used [25]. Besides its overall score, it has 3 subscales that have been assessed individually: system usefulness, information quality, and interface quality. All scales have a maximum possible score of 7, with lower numbers indicating better usability [25]. Effectiveness in the context of this study refers to the ability of MotherHeat Alert to change knowledge and awareness of heat-health risks [26]. This was assessed through comparisons between end point and baseline scores. Effectiveness was therefore evaluated based on (1) heat-protective behavior change, for instance, through the question “Thinking about the last day when it was really hot, what did you do to keep yourself cool?” and (2) knowledge increase on heat and health following the intervention period, for instance, through the question “From your use of the app, do you think that your knowledge about the effects of heat on your health has increased?” Table S1 in Multimedia Appendix 1 provides a visual illustration of the usability and effectiveness concepts and how these were assessed. All analyses were conducted using SPSS version 30.0 (IBM Corp).

### Statistical Analysis

Most self-reported usability and knowledge increase perceptions were assessed at end point, using descriptive statistics. Binary logistic generalized estimating equations (GEE) models were used on 2 questionnaire items, namely “Thinking about the last day when it was really hot, what did you do to keep yourself cool?” and “Thinking about the last day when it was really hot, what did you do to protect your baby from the heat?” to assess whether a significant behavior change was detected by comparing baseline and end point results. We further used 2 methods to assess the in-app survey items: a generalized linear mixed model (GLMM) to evaluate survey items with large discrepancies in repeated responses and a linear mixed effects model with random intercepts, which accounts for substantial between-individual differences at baseline (Table S1 in Multimedia Appendix 1 provides further details regarding choice of statistical model and time of data collection).

### The Heat Health EWS: MotherHeat Alert

The MotherHeat Alert app is a mobile EWS for Android (Google LLC) and iOS (Apple Inc.) users that provides real-time, localized heat warnings with tailored health advice to improve awareness of heat-related health risks and promote protective behavior [26]. The app served as an intervention that integrates environmental data (UTCI temperature, air temperature, humidity, solar radiation, and wind) with the user’s gestational status to deliver personalized alerts and recommend specific, locally adapted protective actions (refer to Figure 1 for app interface). It used a 7-day UTCI daily forecast and issued push notifications at 6 AM whenever at least one of the following 7 days was predicted to exceed the predefined heat alert threshold of 27.3°C UTCI. Individual parameters including age and BMI could also be added to the EWS to further personalize messaging. Participants selected profiles, choosing between pregnancy or postpartum stages, which in turn determined the content and timing of heat alerts and messages. At a minimum, the EWS aimed to increase heat risk awareness and support behavior change, such as using specific cooling techniques, more suitable clothes, and how to stay hydrated, among others, during hot, high-risk periods. In this study, the EWS app was provided free of charge and delivered response messages with locally applicable guidance in local languages.

**Figure 1:**
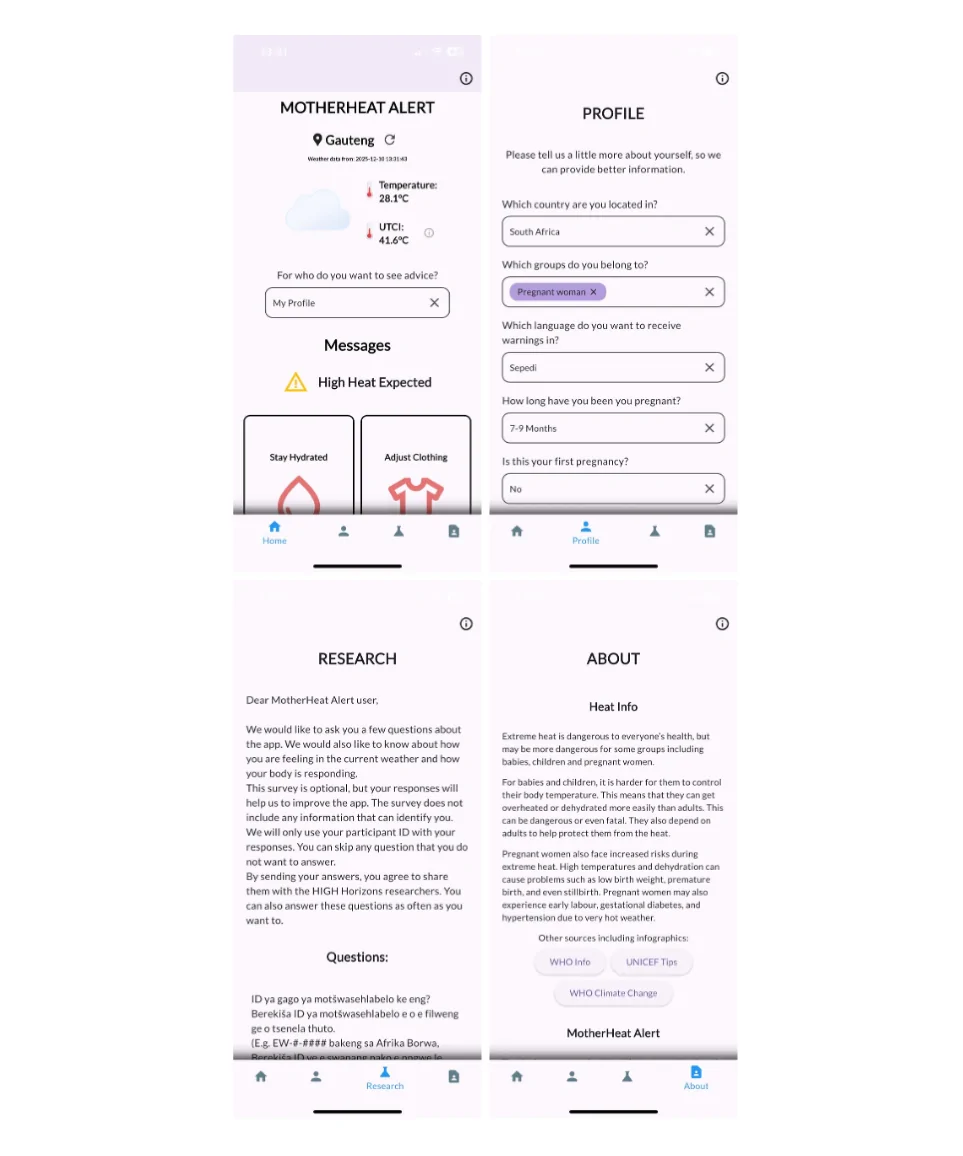
The MotherHeat Alert interface. From left to right, top to bottom: the home screen, the profile screen, the research screen, and the about screen.

### Patient and Public Involvement

A co-design process was used to develop the messages used in the MotherHeat Alert app. This included photovoice workshops with pregnant and postpartum women recruited from the study site to develop messages based on their lived experiences [27]. The draft messages were then presented to community health workers based at the CHC in 2 focus group discussion sessions to enable triangulation and further contextual validation. Further refinements were made in consultation with expert reviewers. This process helped ensure that the EWS was grounded in lived experiences while being informed by professional expertise.

### Ethical Considerations

Both the University of Witwatersrand Human Research Ethics Committee (HREC) in South Africa (approval number 240606) and district approval were granted to conduct the study in a Tshwane health facility. The study was registered with the South African National Clinical Trial Registry (DOH-27-032025-5623), as part of the European Union (EU) and United Kingdom–funded HIGH Horizons project. All participants provided written informed consent. Data were fully anonymized or pseudonymized. Each participant was assigned a unique participant identification number. Participants were reimbursed ZAR400 (±US $25) for attending each study visit.

## Results

### Overview

In total, 280 women were screened for eligibility. Of these, 209 (74.6%) met the inclusion criteria and provided informed consent. Reasons for nonenrollment include not having compatible smartphones (26/280, 9.3%), not planning to remain in the area for 6 months (14/280, 5%), or not willing and/or able to provide written consent (51/280, 18.2%; note that reasons are not mutually exclusive). Of the 209 women who were enrolled, 8 discontinued early: 5 were unreachable after enrollment, 1 declined to install the app, and 2 withdrew after enrollment without providing a reason. Ultimately, 201 participants (100 pregnant and 101 postpartum) were included in the analyses; however, 25 (12.4%) did not complete the midpoint visit, and 50 (24.9%) did not complete the end point visit. Enrollments occurred between October 2024 and January 2025, while 2-month follow-up (midpoint) and 6-month follow-up (end point) visits were conducted between January 2025 and March 2025 and June 2025 and August 2025, respectively. For the pregnant cohort, the number of participants retained was 100 at baseline, 93 at midpoint, and 70 at end point. For the postpartum cohort, 101 were included at baseline, 83 at midpoint, and 82 at end point. Due to the privacy-by-design architecture of the system, alert generation was not recorded on the central server; therefore, the number and timing of alerts displayed to individual participants could not be determined.

At baseline, the median age for both pregnant and postpartum participants was 29 (IQR pregnant women 25-34; IQR postpartum women 24.5-33) years. Most participants completed high or secondary school (117/201, 58.2%), and half were unemployed but looking for work (100/201, 49.8%). Most participants (193/201, 96.0%) reported owning a phone that was not shared with other family members. Almost all (n=200) participants reported using apps on their phone, mostly for social media (198/201, 98.5%) and messaging (92/201, 45.8%; Table 1). Furthermore, 55 of 201 (27.4%) participants reported having a pregnancy or baby monitoring app on their phone, and 79 of 201 (39.3%) participants received pregnancy or baby growth and development messages on their phones (eg, MomConnect). At baseline, prior to the MotherHeat Alert intervention, the largest proportion of participants reported never discussing heat-related health concerns with the community (96/201, 47.7%; Table 2), and only a few participants mentioned knowing about any community programs or public health messages about heat safety (9/201, 4.5%; Table 2). Among the postpartum cohort, very few participants reported knowing about the effect of heat on themselves during pregnancy or the postnatal period (10/101, 9.9%) and their baby (9/101, 8.9%; Table 2). Around half of the participants (102/201, 50.7%) reported that it is hotter inside compared to outdoors (Table 2). A large proportion (65/201, 32.3%) reported wanting to take additional particular heat-protective measures but were unable to do so.

**Table 1:** Descriptive statistics on education, employment, phone ownership, and general app usage at baseline.

Characteristics			Pregnant, n (%)		Post partum, n (%)
Maternal age (years)					
	≤20	0 (0)		5 (5)	
	21-25	23 (23)		18 (17.8)	
	26-30	30 (30)		31(30.7)	
	31-40	23 (23)		33 (32.7)	
	>40	24 (24)		14 (13.9)	
Education					
	Completed only primary school	1 (1)		0 (0)	
	Some high or secondary school	17 (17)		19 (18.8)	
	Completed high or secondary school	54 (54)		63 (62.4)	
	Some tertiary, university, or college	20 (20)		8 (7.9)	
	Completed tertiary, university, or college	8 (8)		11 (10.9)	
	Total	100 (100)		101 (100)	
Employment^a^					
	Employed full-time	35 (34.4)		28 (27.7)	
	Employed part-time	10 (9.8)		3 (3)	
	Self-employed	7 (6.9)		0 (0)	
	Not employed but looking for work	42 (41.2)		58 (57.4)	
	Not employed and not able to work	0 (0)		7 (6.9)	
	Not employed and not looking for work	2 (2)		0 (0)	
	Homemaker	5 (4.9)		5 (5)	
	Student	1 (1)		0 (0)	
	Total	102 (100)		101 (100)	
Phone ownership and app usage^a^					
	Phone used solely by participant	96 (96)		97 (96)	
	Phone shared with other family members	4 (4)		4 (4)	
	Using apps on phone	100 (100)		100 (99)	
	Messaging	39 (39)		53 (52.5)	
	Health	16 (16)		20 (19.8)	
	Weather	30 (30)		35 (33.7)	
	News	9 (9)		15 (14.9)	
	Social media	98 (98)		100 (99)	
	Other	3 (3)		2 (2)	

^a^Notice that this does not add up to the total number of participants per cohort, since response options were not mutually exclusive.

**Table 2:** Self-reported indoor heat-health parameters by pregnant and postpartum cohort at baseline prior to installation of the app.

Participants’ heat-health information and awareness prior to app installation		Pregnant (n=100), n (%)	Postpartum (n=101), n (%)
Discuss health concerns with community			
	Never	46 (46)	50 (49.5)
	Rarely	3 (3)	6 (5.9)
	Sometimes	41 (41)	28 (27.7)
	Often	5 (5)	11 (10.9)
	Always	5 (5)	6 (5.9)
Availability of heat-health information and protective measures			
	Community programs or public health messages about heat safety in the area	4 (4)	5 (5.0)
	Protective action that you would have liked to do, but were unable to	26 (26)	39 (38.6)
Heat-health knowledge postpartum			
	Have you heard about how hot weather might affect your health?	—^a^	10 (9.9)
	Have you heard about how hot weather might affect your baby’s health?	—	9 (8.9)
Indoor heat perception			
	Indoor is cooler	38 (38)	46 (45.5)
	Indoor is hotter	49 (49)	53 (52.9)
	Just as hot inside as outside	10 (10)	2 (2)
	Do not know	3 (3)	0 (0)

^a^Not available.

### Usability

With lower PSSUQ scores indicating better usability, the summed overall score of PSSUQ (questions Q1-Q16) in the pregnant cohort ranged between 1 and 3.44 (mean 1.76, SD 0.60; n=70) and 1 and 2.85 (mean 1.37, SD 0.45; n=81) in the postpartum cohort (Figure 2). The PSSUQ score on system usefulness (Q1-Q6) ranged between 1 and 2.83 (mean 1.34, SD 0.51; n=70) for the pregnant cohort and 1 and 2.83 (mean 1.28, SD 0.39; n=81) for the postpartum cohort. The PSSUQ score on information quality (Q7-Q12) was higher for the pregnant cohort and ranged between 1 and 5.33 (mean 2.52, SD 1.19; n=70) but lower for the postpartum cohort, ranging from 1 to 3.67 (mean 1.48, SD 0.64; n=81). Finally, the PSSUQ score on interface quality (Q12-Q16) ranged between 1 and 4.0 (mean 1.31, SD 0.63; n=70) for pregnant and 1 and 3.25 (mean 1.35, SD 0.53; n=81) for the postpartum cohort (Figure 3). To account for the large dropout rate across the study (Figure 4), we conducted a sensitivity analysis for the total usability-related scores, comparing baseline to the end point. Overall, 2 different scenarios were assessed, one assuming that dropouts occurred due to low usability ratings and the other assuming that dropouts occurred due to other external reasons.

Baseline comparisons between participants who dropped out and those who remained in the study showed no significant difference in age (independent-samples 2-tailed *t* test), self-rated general health, presence of chronic health conditions, perceived indoor heat, housing type (formal vs informal dwelling), or perceived seriousness of heat for themselves or their infants (chi-square tests; all *P*<.05). There was some indication of differences by educational attainment, with a nonsignificant overall association (chi-square test, *P*=.09), but evidence of a significant linear trend (*P*=.02), suggesting lower dropout rates among participants with higher levels of education. Furthermore, sensitivity analyses were conducted to evaluate the effect of missing end point usability data under alternative assumptions. In the worst-case scenario, dropouts were assigned the maximum PSSUQ score of 7, representing the poorest usability and assuming that withdrawal was related to poor user experience. In the as-usual scenario, dropouts were assigned scores comparable to those who completed end point assessment, assuming that dropouts occurred for reasons unrelated to usability. Results were robust under the as-usual assumption (mean 1.53, SD 0.52, 95% CI 1.46-1.61; n=201). Under the worst-case assumption, the mean score was higher (mean 2.91, SD 2.41, 95% CI 2.57-3.24; n=201), indicating poorer usability and greater variability; however, it did not change the overall interpretation of the findings.

For the in-app question “Was the message you received relevant for your situation” (yes or no), current UTCI significantly predicted the likelihood of participants finding messages relevant: for every 1°C increase, the odds of reporting that a message was relevant increased by ±5% (odds ratio [OR] 1.05, 95% CI 1.01-1.09). The model highlighted significant between-person variability in baseline relevance rating (Var=3.59; *P*<.001). For the in-app question “On a scale of 1 to 5, please rate how easy you have found it to use the app” (1=easy, 5=difficult), a comparison of novice users with experienced users assessing whether the perceived ease of use changed with experience showed that the effect of time was statistically significant (*F*_1,6335_=76.18; *P*<.001), indicating that perceived difficulty decreased as participants used the MotherHeat Alert over time. Importantly, however, this statistically significant change was very small in magnitude (marginal *R*^2^=0.007), where most variation can be explained by the differences across users (conditional *R*^2^=0.63). This suggests that 0.7% of the variance in difficulty can be explained through the repeated use over time.

**Figure 2:**
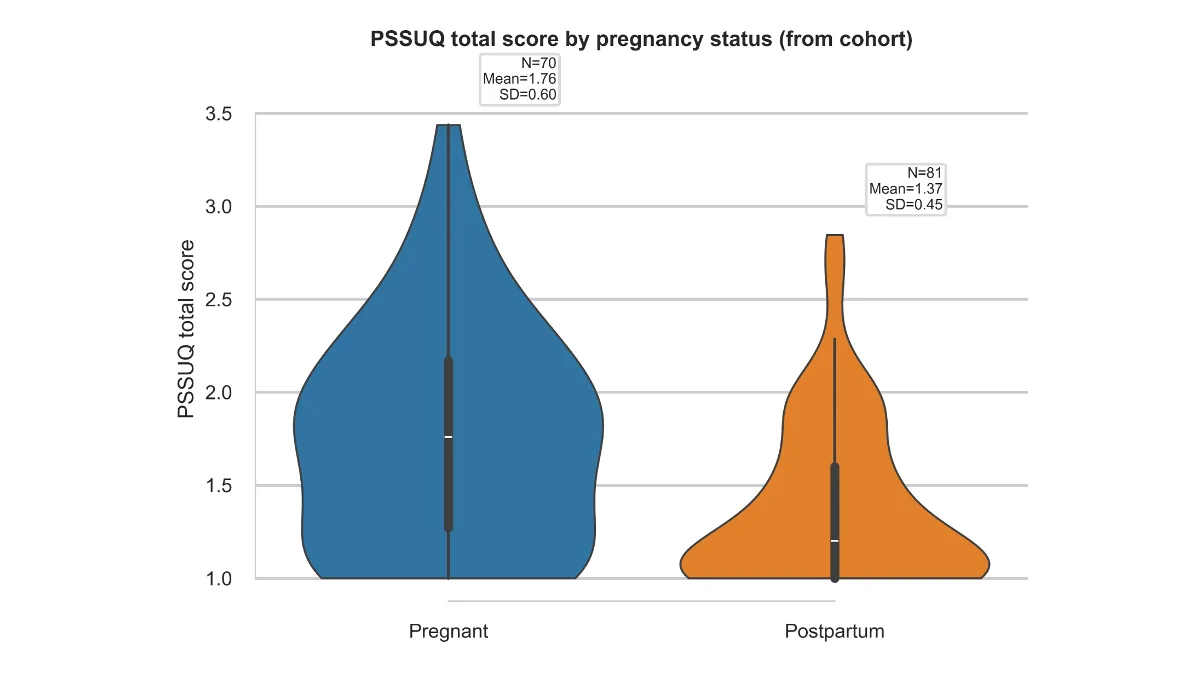
Violin plot showing Post-Study System Usability Questionnaire total score (Q1-16) stratified by pregnant and postpartum cohort. PSSUQ: Post-Study System Usability Questionnaire.

**Figure 3:**
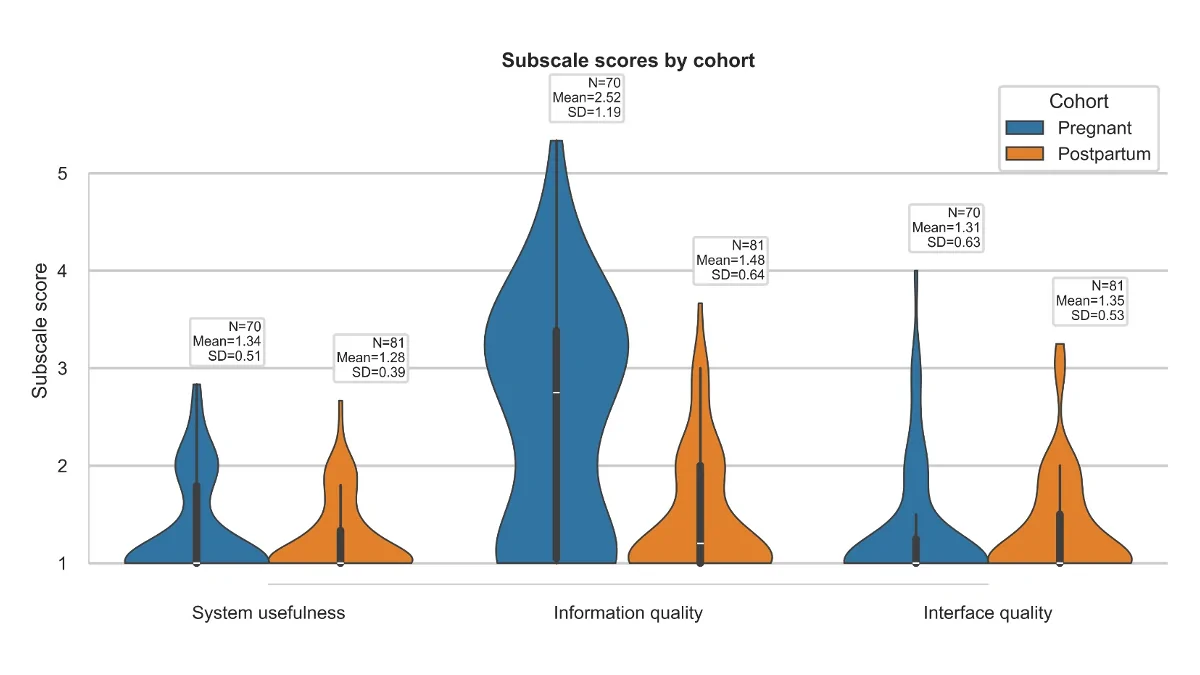
Violin plot comparing the PSSUQ subscales, system usefulness, information quality and interface quality. Results are stratified amongst pregnant and postpartum cohorts. PSSUQ: Post-Study System Usability Questionnaire.

**Figure 4:**
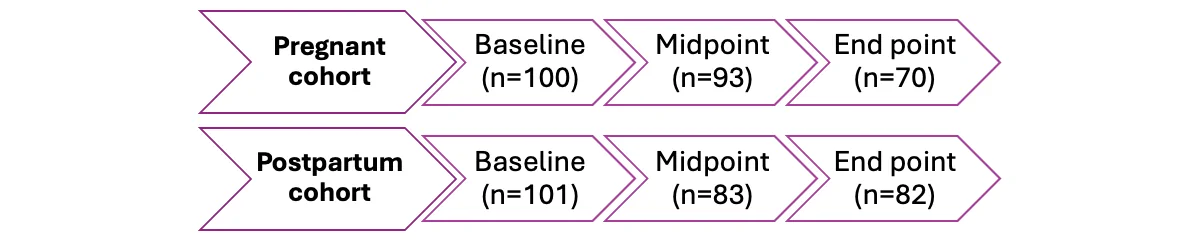
Dropout rate across the study; at baseline, midpoint (2-month follow up) and end point (6-month follow up) for pregnant cohort and postpartum cohort.

### Effectiveness

#### Knowledge Increase

The largest proportion in both pregnant and postpartum cohorts self-reported a large to very large knowledge increase (55/70, 78.6% and 72/82, 87.8%, respectively) on the effects of heat on pregnant women’s health (Table 3). The postpartum cohort also indicated a large to very large knowledge increase (72/82, 87.8%) on the self-reported effects of heat on the baby’s health.

**Table 3:** Self-reported knowledge increase due to early warning systems app reported by pregnant and postpartum cohort.

Self-reported knowledge increases due to EWS^a^ use			Values
Pregnant cohort: effects of heat on pregnancy health, n (%)			
	No-slight increase	2 (2.8)	
	Moderate increase	13 (18.6)	
	Large-very large increase	55 (78.6)	
	Do not know	0 (0)	
	Total	70 (100)	
Postpartum cohort: effects of heat on infants’ health, n (%)			
	No-slight increase	0 (0)	
	Moderate increase	9 (11)	
	Large-very large increase	72 (87.8)	
	Do not know	1 (1.2)	
	Total	82 (100)	

^a^EWS: early warning systems.

#### Behavior Change

The item assessing “Thinking about the last day when it was really hot, what did you do to keep yourself cool?” contained 17 potential response options. In a GEE model, after adjusting for maternal age and education, a significant behavior change was noted for 8 options comparing end point (6-month follow-up after app introduction) to baseline (preapp introduction) in paired analyses (Table 4). For both pregnant (n=70) and postpartum cohorts (n=82), the following behaviors showed statistically significant shifts toward less frequent use after introduction of the app: drinking more water, drinking more fluids, bathing more, using more light and loose clothing, opening windows, and wearing a hat. For the pregnant women cohort, eating more fruits and increased use of a fan also decreased significantly between baseline and end point. For the postpartum cohort, working less and staying indoors decreased significantly between baseline and end point. A total of 4 response options—using Mcako or clay or ibovu (a type of clay that is high in minerals and traditionally used as skin protection from sun exposure), air conditioning, using an umbrella, and did nothing—could not be analyzed due to an insufficient number of cases across groups for statistical testing. Importantly, all the comparisons between baseline and end point indicate a negative change, highlighting that less protective heat action had been taken at end point compared to baseline (Table 4). Comparing midpoint (2-month follow-up after app introduction) to baseline, a similar, yet less pronounced trend was discerned, with 2 response options showing a significant decrease in protective behavior in the pregnant cohort, including using a fan and wearing light and loose clothing. In the postpartum cohort, 7 behaviors indicated a significant decrease, including drinking other fluids, working less, bathing more frequently, wearing light and loose clothing, opening windows, wearing a hat, and staying indoors (Table S2 in Multimedia Appendix 1). In this GEE model, 2 covariates were controlled for: maternal age and education [28-31] since both have consistently been associated with more protective health behavior. Effect estimates of a stratified analysis of these covariates can be found in Table S3 in Multimedia Appendix 1 for end point results and Table S4 in Multimedia Appendix 1 for midpoint results. Statistical significance of the correlations did not differ between crude and adjusted models. At end point, almost all participants from the pregnant (66/69, 96%) and postpartum (72/82, 88%) cohorts indicated that these measures were taken because they were alerted by the app.

The item “Thinking about the last day when it was really hot, what did you do to protect your baby from the heat?” contained 13 answer options, where giving the baby more water, giving the baby more fruit, and seeking shaded or cool areas yielded a positive behavior change, whereas dressing the baby in light and loose clothing and opening windows showed a decrease at end point in the adjusted model (Table 5). Again, the midpoint results yielded a similar pattern, where seeking shaded or cool areas and other measures showed an increase in this adaptive behavior, and dressing the baby in light and loose clothing, opening windows, and keeping the baby indoors showed a decrease in this behavior in the adjusted model (Tables S5 and S6 in Multimedia Appendix 1). Most postpartum women (72/82, 87.8%) indicated that these protective measures were taken because they were alerted by the app. The largest proportion of self-reported behavior change due to the alerts received through the app rated this change as large to very large: 67.2% (47/70) in the pregnant cohort and 82.9% (68/82) in the postpartum cohort (Table S7 in Multimedia Appendix 1).

The in-app question “Did you follow the advice provided? (yes or no),” which was linked to current UTCI, significantly predicted the likelihood of following advice; for every 1°C increase in current UTCI, the odds of following advice increased by ±14% (OR 1.14, 95% CI 1.09-1.20). The model highlighted significant between-person variability in the baseline following advice rating (random intercept var=5.072; *P*<.001).

**Table 4:** Pregnant and postpartum effect estimates from a generalized estimating equation assessing how pregnant and postpartum women kept themselves cool during hot days, comparing end point to baseline results by asking “Thinking about the last day when it was really hot, what did you do to keep yourself cool?”

Behavior^a^	Pregnant				Postpartum		
	Baseline (n=100), n	End point (n=70), n	aOR^b^ (95% CI)^c^	Baseline (n=101), n		End point (n=82), n	aOR (95% CI)
Drank more water	95	66	0.09 (0.04-0.23)	+91		78	0.37 (0.16-0.86)
Drank other fluids	17	8	0.42 (0.18-0.97)	72		51	0.40 (0.23-0.72)
Ate more fruits	17	8	0.42 (0.19-0.92)	41		42	1.04 (0.64-1.70)
Sought shaded or cool areas	21	15	0.65 (0.32-1.33)	71		65	0.75 (0.46-1.23)
Went to cooler buildings	12	12	1.00 (0.41-2.42)	40		39	0.96 (0.57-1.62)
Used Mcako^d^/clay/ibovu	2	0	—^e^	1		6	6.34 (0.73-55.10)
Worked less	15	17	1.16 (0.58-2.30)	70		49	0.40 (0.25-0.65)
Bathed more frequently	36	23	0.53 (0.28-0.99)	82		68	0.47 (0.25-0.90)
Used fan	44	15	0.22 (0.11-0.42)	48		42	0.78 (0.47-1.31)
Used air conditioning	6	0	—	8		10	1.28 (0.49-3.37)
Wore light and loose clothing	36	24	0.56 (0.32-0.99)	93		72	0.20 (0.09-0.46)
Opened windows	50	37	0.58 (0.35-0.98)	98		68	0.06 (0.02-0.19)
Put feet or hands in cool water	8	4	0.48 (0.15-1.52)	34		24	0.61 (0.36-1.05)
Wore a hat	26	8	0.25 (0.10-0.58)	76		62	0.51 (0.29-0.88)
Used umbrella	41	0	—	79		0	—
Stayed indoors	40	52	1.64 (0.95-2.84)	74		58	0.47 (0.26-0.84)
Did nothing	0	0	—	0^e^		0	—

^a^Adjusted for maternal age and education.

^b^aOR: adjusted odds ratio.

^c^Model having insufficient data to execute the analysis.

^d^Mcako is a type of clay high in minerals that is traditionally used as skin protection from sun exposure.

^e^Not available.

**Table 5:** Postpartum cohort generalized estimating equation effect estimates assessing how mothers kept their babies cool during hot days, comparing end point to baseline results by asking: “Thinking about the last day when it was really hot, what did you do to protect your baby from heat?”

Behavior^a^	Baseline (n=101), n	End point (n=82), n	OR^b^ (95% CI)
Breastfed more frequently	57	58	1.04 (0.64-1.70)
Gave baby more water	55	77	2.73 (1.57-4.75)
Gave baby more fruits	17	27	1.86 (1.05-3.28
Used Mcako^c^/clay/ibovu	0	0	—^d^
Sought shaded/cool areas	43	59	1.96 (1.17-3.28)
Bathed baby more frequently	68	67	0.96 (0.53-1.72)
Used fan	17	23	1.49 (0.73-3.03)
Used air conditioning	3	2	—
Dressed baby in light and loose clothing	91	74	0.29 (0.14-0.63)
Opened windows	86	69	0.37 (0.18-0.73)
Kept baby indoors	73	64	0.65 (0.35-1.20)
Did nothing	0	0	—
Other	2	12	6.69 (1.42-31.63)

^a^Adjusted for covariates: maternal age and education.

^b^OR: odds ratio.

^c^Mcako is a type of clay high in minerals that is used as skin protection or often also ingested.

^d^Not applicable; represents a model having insufficient data to execute the analysis.

## Discussion

### Principal Findings

This study assessed the usability and effectiveness of an EWS aimed at reducing heat-related health risks among pregnant and postpartum women.

Usability results indicated that the MotherHeat Alert was considered useful and was well accepted by users. The effectiveness findings of the MotherHeat Alert suggest that engagement with the EWS led to self-reported increases in adoption of heat-protective knowledge and behavior. Comparisons of actual self-reported heat-protective behaviors at baseline and end point showed that these behaviors largely decreased by the end of the 6-month intervention period. This decrease could also be identified to a lesser extent at the 2-month midpoint results. Importantly, these results should be interpreted with caution, as midpoint and end point assessments were conducted during cooler months, introducing seasonal variation as an important confounder. In addition, approximately one quarter of participants were lost to follow-up between baseline and end point.

### Usability of the MotherHeat Alert

Successful digital health interventions rely on effective integration, adoption, and sustained use by their intended users [32,33]. The Technology Acceptance Model emphasizes perceived usefulness and ease of use as key determinants of technology adoption [34,35]. The positive usability and knowledge outcomes observed in this study support the relevance of these principles to heat-related EWS. Usability, assessed using the PSSUQ questionnaire, was high overall, with system usefulness, information quality, and interface quality all scoring above average compared to norms reported by Lewis and James [25]; the system usefulness subscale scored highest and the information quality subscale lowest. Although similar patterns were observed between pregnant and postpartum cohorts, postpartum women consistently rated the EWS’s usability higher, compared to pregnant women, and a greater variability in responses was typically observed in the pregnant cohort. A total of 2 additional in-app usability indicators, “perceived relevance of warnings” and “ease of use,” showed significant changes over time. The “perceived relevancy of warnings” increased with rising temperatures, with a 5% increase in likelihood per 1°C rise, suggesting that alerts aligned well with users’ lived experiences. “Ease of use” improved over time, indicating that with repeated use and increased familiarity, the EWS was perceived as easier to use, a trend that has been seen in other studies [36,37].

### Effectiveness of the MotherHeat Alert

Most intervention studies in African settings focus on health promotion and disease prevention, aiming to improve health knowledge and encourage behavior change [14]. These generally report positive results [14], which is reflected in the outcomes of this study where women expressed interest in weather apps and general involvement with the EWS. Education and awareness building are among the most common and effective apps of digital health interventions [32]. Behavior change is therefore a suitable measure of effectiveness, as education and knowledge shaping are widely used and effective behavior change techniques [38,39]. High engagement with the EWS supports its effectiveness, as 95.7% (66/69) of pregnant participants and 87.8% (72/82) of postpartum women reported taking protective actions in response to alerts. Effectiveness, measured through self-reported knowledge increase, indicated that at end point, 78.6% (55/70) of pregnant women showed a large to very large knowledge increase on the effects of heat on their own health. For the postpartum cohort, 87.8% (72/82) reported a large to very large knowledge increase of the effects of heat on their own and their baby’s health. The warnings issued through the MotherHeat Alert resulted in preventive actions, suggesting that the recommended measures were practical and feasible for participants to carry out.

Nevertheless, when comparing baseline to end point outcomes, 9 of 17 behaviors reduced significantly among pregnant participants; a similar, but less pronounced trend was observed when comparing midpoint to baseline. Seasonal variation is an important confounder when interpreting the observed decrease in protective behaviors. Baseline assessments were conducted from October 2024 to January 2025, covering the transition period into warmer and early summer months; midpoint assessments from January to March 2025, spanning the later summer period and the early transition into cooler months; and end point assessments from June to August 2025, during the cooler months. Baseline and end point assessments therefore occurred during substantially different climatic conditions, and changes in heat-protective behaviors cannot be attributed to the intervention alone but must be interpreted in the context of seasonal variation. This would also explain why the reduction in heat-protective behavior was less pronounced comparing midpoint to baseline than end point to baseline. As days became cooler, there was no need for pregnant and postpartum women to apply cooling measures as the ambient temperatures were not high enough to warrant adaptive behavior. Fewer cooling behaviors during cooler months should therefore not necessarily be interpreted as poorer adherence or reduced intervention effectiveness but may instead represent an appropriate behavioral response to lower heat exposure. This interpretation is supported by our in-app data, which showed greater adherence to warning advice at higher temperatures, indicating that perceived risk intensifies responsiveness. As the MotherHeat Alert and heat-health apps and EWS more broadly are most relevant during warmer periods, strategies to reengage and retain users ahead of following heat seasons should therefore be considered. Future evaluations should compare behavioral outcomes during similar climatic conditions, for example by conducting baseline and follow-up assessments within the same warm season or across multiple summers for clearer findings and interpretation. For protective behaviors exhibited by postpartum women directed toward infants, a similar negative trend, highlighting less protective behavior at end point, could be identified in some behavior responses, yet a positive trend, indicating more protective behavior, could be seen in others. Among postpartum women, the ability to work less and stay indoors may have been affected by the urgency of returning to employment after childbirth or a need to seek employment as financial responsibilities have increased.

The observed reductions in self-reported protective behaviors should not necessarily be interpreted as indicating reduced engagement with heat-protective practices. Although end point assessments took place during the cooler season, participants were asked to report behaviors undertaken on “the last day when it was really hot”, rather than behaviors undertaken at the time of assessment. During the cooler season, this may have required participants to recall behaviors from further back in time than during the warmer periods, potentially affecting the accuracy of their responses. In addition, social desirability may have influenced self-reported behaviors, particularly at baseline when participants may have been more inclined to report behaviors perceived as desirable or appropriate. Overall, it is further likely that at the start of the intervention, participants were more engaged and motivated to use a new technology, which may have temporarily increased both attention to the app and self-reported adoption of heat-protective behaviors. However, over time, the novelty of participation likely diminished as attention fatigue set in, and actual behaviors may have returned to preintervention patterns, contributing to lower observed heat-protective practices at midpoint and end point. Therefore, the observed changes may reflect a combination of seasonality, social desirability, and reduced motivation over time, rather than an intervention effect alone.

### Limitations

This study has several limitations. Participation was limited to women [14] attending health facilities and owning smartphones, introducing potential selection bias and thereby excluding some of the most vulnerable populations. Convenience sampling through antenatal clinics may have introduced additional selection bias, so findings may not be representative of the broader population of pregnant and postpartum women in South Africa and should therefore be generalized with caution. As the MotherHeat Alert is a text-based EWS, which delivers warnings through written messages, illiterate individuals were likely excluded; literacy rates were not assessed. To increase inclusivity, EWS apps should ideally rely more on graphic messages or audio prompts. Additionally, local infrastructure constraints, such as unstable electricity supply to charge mobile phones, unstable network, shutting down phones to preserve battery power [16], or deleting the app to preserve storage space on end-devices, may have affected notifications, alerts, and system performance. Recruitment in this study was constrained as some women lacked smartphones, while others had devices with limited storage. Installing the app often required deleting photos or other apps, which some participants were unwilling to do. Furthermore, the EWS app required mobile data for daily temperature synchronization and warning delivery. Future EWS should ideally function without the requirement for mobile data or operate through agreements with telecommunication providers to waive data charges (ie, zero-rated app). Finally, all outcomes were self-reported, introducing the possibility of response and social desirability bias. Given the enrollment procedures, participants were aware of project aims, which may have influenced their responses. Furthermore, participant engagement in this study may have been influenced by monetary reimbursement, which may suggest that women in real-world settings may be less likely to install or use the app given these barriers.

Given that the study intended to assess usability and effectiveness over the short- and long-term, 6 months of follow-up was required. This resulted in seasonal timing of data collection becoming a limitation, where baseline data was collected during warmer months, while end point measures occurred during colder months. This may partly explain the decline in reported protective behaviors. Furthermore, given that the proportion of adaptive behaviors reported at baseline was high, particularly among postpartum women, participants may already have adapted to heat exposure, such that explicit warnings added limited new information. Dropout rates in this study were substantial, and usability outcomes were therefore further examined using sensitivity analyses under 2 assumptions: a worst-case scenario, in which participants discontinued due to poor usability, and an as-usual scenario, in which dropouts were unrelated to EWS usability. Baseline characteristics did not differ significantly between participants who dropped out and those who remained, suggesting no clear evidence of systematic differences, thereby supporting the as-usual scenario. In addition, local contextual factors indicate that some participants may have discontinued the follow-up because women may return to their place or country of origin after receiving maternity care in South Africa [40]. It is also important to note that women reported wanting to take additional heat-protective behavior at baseline, but reported they were unable to do so. This finding, however, reflected their general perceived ability to protect themselves from heat, rather than their ability to follow the specific behavioral advice provided through the MotherHeat Alert, which was codeveloped with intended users to ensure that the recommended actions were practical and feasible within their context. Furthermore, the project-based design and limited timeframe restricted conclusions about long-term usability beyond the novelty effect of a new intervention, and about sustainability and scalability, which should be prioritized prior to consideration for mass roll-out. This structural limitation presents a common limitation of intervention studies in African contexts [14]. Evidence on cross-country uptake of the EWS, scalability, and continuity of digital health interventions in Africa remains weak, with most existing studies limited to pilot or small-scale implementations [14,34]. Finally, it should be noted that, to the best of our knowledge, the PSSUQ has not yet been validated in South Africa, although previous research has used the PSSUQ in South Africa [41] and other African countries [42].

### Future Directions

Given the borderless nature of heat risk and its increasing severity, the development of a global heat EWS should be a long-term goal [8]. Despite their potential, few countries have heat-specific EWS, and no global system exists for the specific vulnerable groups. This represents a critical gap, particularly since the United Nations’ Early Warning for All initiative aims to ensure effective EWS coverage by 2027 [43]. Currently, South Africa still issues heat warnings using absolute air temperature thresholds unrelated to health responses [44]. Future systems should be built around specific heat-health indicators, locally relevant, and integrated heat stress thresholds and codeveloped communication strategies. Greater attention is needed to gendered and intersecting vulnerabilities to ensure access to warnings [15]. Future research should prioritize rigorous impact evaluations, cost-effectiveness analyses, and qualitative studies on implementation context [14,32]. In addition, simplicity of design and modest objectives have repeatedly been associated with successful implementation in African contexts, since these products were least affected by contextual challenges [14]. Greater emphasis is needed on integrating the digital interventions into the existing health care infrastructure and addressing scalability [45]. Given the constraints of limited memory on mobile phones, together with the high use of social media among participants, stand-alone apps for heat-health warnings may not be the most efficient approach. Integrating these warnings into social media platforms may therefore be a more feasible and effective strategy.

Digital health interventions are generally received with optimism [14]. As mobile phone use is rapidly increasing in sub-Saharan Africa, the digital health technologies designed for this context follow suit [34]. Although evidence on long-term effectiveness and impact remains limited, this study demonstrates high effectiveness and usability of a heat-related EWS. Education and training have shown to reduce heat stress and illness [39], as shown in a study where digital technologies supporting maternal health in sub-Saharan Africa have contributed to improved maternal and infant health decision-making [16].

### Conclusion

Overall, our study strengthens the growing evidence that well-designed, user-centered digital health technologies, including heat EWS, have high usability outcomes and contribute to knowledge gains in the context of climate-related health risks. However, the contribution of EWSs to improved health outcomes in broader contexts will depend on whether they are perceived as useful, easy to use, and whether social, cultural, and policy contexts have been considered during their implementation. This study demonstrates the potential of a co-designed heat-health EWS tailored toward pregnant and postpartum women to support protective responses to heat exposure. The intervention demonstrated high self-rated usability and perceived effectiveness among users. However, the observed decrease in heat-protective behaviors when comparing reported behavior at baseline compared to end point assessments should be interpreted with caution, as seasonal variation was linked to the follow-up visits through the study design, making it impossible to attribute behavioral changes to the intervention alone. Digital health technologies may play an important role in strengthening climate adaptation responses for maternal health. Focusing on the usability and effectiveness of EWS interventions, considering contextual relevance, assessing long-term usability beyond the novelty effect, and ensuring access for everyone by integrating these interventions into existing health systems is required to achieve this potential.

## Appendix

Multimedia Appendix 1 Supplementary tables, figures, and questionnaire items related to the usability and effectiveness of the MotherHeat Alert Early Warning System.

## Abbreviations

CHC: community health center
EU: European Union
EWS: early warning system
GEE: generalized estimating equations
GLMM: generalized linear mixed model
HIGH: Heat Indicators of Global Health
HREC: Human Research Ethics Committee
OR: odds ratio
PSSUQ: Post-Study System Usability Questionnaire
UTCI: Universal Thermal Climate Index

## Data Availability

The datasets generated and analyzed during this study are not publicly available due to sensitive health data and participant confidentiality but are available from the corresponding author on reasonable request.
