# Supplementary material for: Mobile Heat Early Warning System for Pregnant and Postpartum Women in South Africa: Prospective Observational Evaluation Study
Source: JMIR Mhealth Uhealth. 2026 Sep 25;14:e102863. doi: 10.2196/102863 (PMC13653204; doi:10.2196/102863)
Supplement: Supplementary file 1 [file mhealth_v14i1e102863_app1.docx]

**Multimedia Appendix 1: Supplementary tables, figures and questionnaire items related to the usability and effectiveness of the MotherHeat Alert Early Warning System**

***Table 1***. Analyses performed to assess the effectiveness and usability of the EWS MotherHeat Alert in South Africa.

*To see whether temperature significantly impacts whether messages are deemed relevant

| **Construct** | | **Item** | **Analysis** | **Measure** | **Statistical method** |
| --- | --- | --- | --- | --- | --- |
|  | **Behaviour change** | Thinking about the last day when it was really hot, what did you do to keep yourself cool? | baseline-endline | Binary (ticked= 1, not ticked= 0) | Generalised estimating equation |
|  |  | Did you take any of these measurements because you were alerted by the app | endline | Binary (yes= 1, no= 0) | descriptive statistics |
|  |  | Thinking about the last day when it was really hot, what did you do to protect your baby from the heat? | baseline-endline | Binary (ticked= 1, not ticked= 0) | Generalised estimating equation |
|  |  | Did you take any of these measures because you were alerted by the app | endline | Binary (yes= 1, no= 0) | descriptive statistics |
|  |  | Do you think you have changed your behaviour (or the way you do things) during hot days because of alerts or messages you received from the app? | endline | 1-5 scale, 1 no change, 2 slight change, 3 moderate change, 4 large change and 5 very large change | descriptive statistics |
|  |  | Did you follow the advice provided? | continuous | correlated with current UTCI* | Generalised linear mixed model |
|  | **Knowledge increase** | From your use of the app, do you think that your knowledge about the effects of heat on your health has increased? | endline | 1-5 scale, 1 no increase, 2 slight increase, 3 moderate increase, 4 large increase and 5 very large increase | descriptive statistics |
|  |  | From your use of the app, do you think that your knowledge about the effects of heat on your baby’s health has increased? | endline | 1-5 scale, 1 no increase, 2 slight increase, 3 moderate increase, 4 large increase and 5 very large increase | descriptive statistics |
|  | **PSSUQ summed** | Q1-Q16 | endline | Scale 1-7, strongly agree (1) to strongly disagree (7) | descriptive statistics |
|  | **PSSUQ system usefulness** | Q1-Q6 | endline | Scale 1-7, strongly agree (1) to strongly disagree (7) | descriptive statistics |
|  | **PSSUQ information quality** | Q6-12 | endline | Scale 1-7, strongly agree (1) to strongly disagree (7) | descriptive statistics |
|  | **PSSUQ interface quality** | Q12-16 | endline | Scale 1-7, strongly agree (1) to strongly disagree (7) | descriptive statistics |
|  | **Ease of use** | On a scale of 1 to 5, please rate how easy you have found it to use the app | continuous | compare earlier to later responses, difficulty scale 1-5: easy (1) to difficult (5) | Linear mixed-effects model |
|  | **Relevance** | Was the message you received relevant for your situation? | continuous | correlated to current UTCI** | Generalised linear mixed model |

***Table 2.*** Pregnant and postpartum effect estimates from generalised estimating equation assessing how **pregnant and postpartum women kept themselves cool** during hot days comparing **midline to baseline** results. Adjusted for covariates: maternal age and education. Na represents model having insufficient data to execute the analysis.

*Mcako is type of clay high in minerals that is used as skin protection or often also ingested

| Behaviour | n baseline | n midline | p | OR | 95% CI | n baseline | n midline | p | OR | 95% CI |
| --- | --- | --- | --- | --- | --- | --- | --- | --- | --- | --- |
| pregnant | | | | | | postpartum | | | | |
| Drank more water | 95/100 | 98/93 | .523 | 0.69 | 0.22 - 2.18 | 91/101 | 81/83 | 0.62 | 0.44 | 0.19 - 1.04 |
| Drank other fluids | 17/100 | 13/93 | .416 | 0.73 | 0.36 - 1.57 | 72/101 | 57/83 | **.002** | 0.40 | 0.23 - 0.72 |
| Ate more fruits | 17/100 | 13/93 | .432 | 0.73 | 0.33 - 1.61 | 41/101 | 41/83 | 1.00 | 1.00 | 0.63 - 1.59 |
| Sought shaded/cool areas | 21/100 | 17/93 | .450 | 0.78 | 0.39 - 1.52 | 71/101 | 70/83 | .853 | .95 | 0.56 - 1.62 |
| Went to cooler buildings | 12/100 | 7/93 | .255 | 0.55 | 0.20 - 1.54 | 40/101 | 42/83 | .752 | 1.09 | 0.65 - 1.81 |
| Used Mcako*/clay/ibovu | 2/100 | 1/93 | na | na | na | 1/101 | 2/83 | na | na | na |
| Worked less | 15/100 | 7/93 | .077 | 0.42 | 0.16 - 1.10 | 70/101 | 51/83 | **<.001** | 0.43 | 0.27 - 0.71 |
| Bathed more frequently | 36/100 | 27/93 | .114 | 0.65 | 0.39 - 1.11 | 82/101 | 65/83 | **.004** | 0.42 | 0.23 - 0.75 |
| Used fan | 44/100 | 30/93 | **.025** | 0.53 | 0.31 - 0.93 | 48/101 | 37/83 | .053 | 0.62 | 0.39 - 1.01 |
| Used air conditioning | 6/100 | 4/93 | na | na | na | 8/101 | 6/83 | .564 | 0.72 | 0.24 - 2.18 |
| Wore light & loose clothing | 36/100 | 18/93 | **.002** | 0.38 | 0.21 - 0.69 | 93/101 | 72/83 | **<.001** | 0.20 | 0.09 - 0.46 |
| Opened windows | 50/100 | 40/93 | .164 | 0.66 | 0.37 - 1.18 | 98/101 | 74/83 | **<.001** | 0.08 | 0.02 - 0.29 |
| Put feet / hands in cool water | 8/100 | 5/93 | 0.408 | 0.60 | 0.18 - 1.99 | 34/101 | 25/83 | .136 | 0.64 | 0.36 - 1.15 |
| Wore a hat | 26/100 | 17/93 | .118 | 0.58 | 0.29 - 1.15 | 76/101 | 56/83 | **.003** | 0.38 | 0.20 - 0.72 |
| Used umbrella | 41/100 | 0/93 | na | na | na | 79/101 | 0/83 | na | na | na |
| Stayed indoors | 40/100 | 29/93 | 0.10 | 0.61 | 0.34 - 1.10 | 74/101 | 60/83 | **.043** | 0.52 | 0.28 - 0.82 |
| Did nothing | 0/100 | 0/93 | na | na | na | 0/100 | 10/83 | na | na | na |

***Table 3****. Overall group covariate* *Wald χ² statistic with associated significance value assessing the question: Thinking about the last day when it was hot what did you do to protect* ***yourself*** *from the heat?, comparing* ***endline to baseline****. Education covariate was adjusted due to too few observations in each category to the following: 1* = some primary school, completed primary school, some high / secondary school, 2 = completed high / secondary school, 3 = some tertiary / university / college education, completed tertiary / university / college education. Highlighted in bold are the results that are significant on an overall group covariate level. Na indicates when model has too few observations to run the analysis.*

**Education category 1 serves as reference category.*

| **Behaviour** | **Covariate** | **Beta** | **p** | **Beta** | **p** |
| --- | --- | --- | --- | --- | --- |
|  |  | **pregnant** | | **postpartum** | |
| Drink more water | Maternal Age | -0.040 | .285 | -0.031 | .339 |
|  | Education (cat 2) | -1.286 | **.011** | -0.126 | .788 |
|  | Education (cat 3) | -1.507 | **.007** | -1.072 | .124 |
| Drink other fluids | Maternal Age | 0.072 | .119 | -0.022 | .412 |
|  | Education (cat 2) | -0.347 | .530 | -0.209 | .611 |
|  | Education (cat 3) | 0.250 | .698 | -0.410 | .416 |
| Ate more fruits | Maternal Age | -0.032 (1) | .427 | -0.034 | .195 |
|  | Education (cat 2) | -0.730 | .272 | -.897 | 0.58 |
|  | Education (cat 3) | -0.095 | .898 | -1.014 | 0.93 |
| Seek shaded areas | Maternal Age | 0.033 | .324 | .0.012 | .688 |
|  | Education (cat 2) | -0.944 | .088 | -0.849 | .056 |
|  | Education (cat 3) | 0.048 | .941 | -1.358 | **.030** |
| Cooler building | Maternal Age | 0.015 | .695 | 0.007 | .769 |
|  | Education (cat 2) | 0.657 | .171 | -0.428 | .300 |
|  | Education (cat 3) | 0.906 | .118 | -0.216 | .664 |
| Used Mcako* | Maternal Age | na | na | 0.022 (1) | .717 |
|  | Education (cat 2) | na | na | -0.114 | .918 |
|  | Education (cat 3) | na | na | -0.681 | .576 |
| Worked less | Maternal Age | 0.006 | .864 | -0.064 | **.037** |
|  | Education (cat 2) | .327 | .551 | 0.197 | .655 |
|  | Education (cat 3) | .234 | .627 | 0.083 | .880 |
| Bathed more | Maternal Age | 0.002 | .936 | -0.024 | .488 |
|  | Education (cat 2) | 0.045 | .919 | 0.175 | .688 |
|  | Education (cat 3) | 0.098 | .840 | -0.385 | .471 |
| Used fan | Maternal Age | -0.044 | .122 | -0.032 | .223 |
|  | Education (cat 2) | -0.376 | .406 | 0.000 | .999 |
|  | Education (cat 3) | -0.769 | .140 | -0.398 | .397 |
| Used air con | Maternal Age | na | na | -0.039 | .243 |
|  | Education (cat 2) | na | na | -0.767 | .311 |
|  | Education (cat 3) | na | na | -0.849 | .317 |
| Wore light and loose clothing | Maternal Age | .003 | .922 | -0.057 | .128 |
|  | Education (cat 2) | -0.455 | .356 | -1.072 | **.032** |
|  | Education (cat 3) | 0.001 | .998 | -1.003 | .098 |
| Opening windows | Maternal Age | -0.022 | .432 | -0.032 | .457 |
|  | Education (cat 2) | 0.042 | .918 | -0.766 | .169 |
|  | Education (cat 3) | -0.353 | .413 | -1.396 | .055 |
| Cold water immersion | Maternal Age | -0.069 | .108 | -0.005 | .868 |
|  | Education (cat 2) | 0.020 | .982 | 0.002 | .997 |
|  | Education (cat 3) | -0.324 | .715 | -0.012 | .984 |
| Wear a hat | Maternal Age | -0.014 | .694 | -0.033 | .287 |
|  | Education (cat 2) | 0.066 | .889 | -1.026 | **.025** |
|  | Education (cat 3) | 0.540 | .325 | -1.443 | **.018** |
| Use umbrella | Maternal Age | .0.031 | .406 | 0.005 | .916 |
|  | Education (cat 2) | -0.892 | .130 | -0.792 | .180 |
|  | Education (cat 3) | -0.360 | .581 | -1.590 | .078 |
| Stay indoors | Maternal Age | -0.040 | .145 | -0.011 | .688 |
|  | Education (cat 2) | -0.543 | .164 | -1.126 | **.005** |
|  | Education (cat 3) | -0.456 | 290 | -1.894 | **<.001** |
| Do nothing | Maternal Age | na | na | na | na |
|  | Education (cat 2) | na | na | na | na |
|  | Education (cat 3) | na | na | na | na |

***Table 4****. Overall group covariate* *Wald χ² statistic with associated significance value assessing the question: Thinking about the last day when it was* ***hot what did you do to protect yourself from the heat****?, comparing* ***midline to baseline****. Education covariate was adjusted due to too few observations in each category to the following: 1* = some primary school, completed primary school, some high / secondary school, 2 = completed high / secondary school, 3 = some tertiary / university / college education, completed tertiary / university / college education. Highlighted in bold are the results that are significant on an overall group covariate level. Na indicates when model has too few observations to run the analysis.*

** Education category 1 serves as reference category.*

| **Behaviour** | **Covariate** | **Beta** | **p** | **Beta** | **p** |
| --- | --- | --- | --- | --- | --- |
|  |  | **pregnant** | | **postpartum** | |
| Drink more water | Maternal Age | 0.100 | .**021** | 0.029 | .366 |
|  | Education (cat 2) | 1.771 | **.027** | 0.354 | .443 |
|  | Education (cat 3) | 0.744 | .310 | .739 | .251 |
| Drink other fluids | Maternal Age | -0.029 | .440 | -0.005 | .865 |
|  | Education (cat 2) | 0.604 | .279 | .132 | .755 |
|  | Education (cat 3) | -0.048 | .940 | .844 | .099 |
| Ate more fruits | Maternal Age | 0.047 | .159 | 0.058 | .062 |
|  | Education (cat 2) | -0.010 | .988 | 1.229 | **.016** |
|  | Education (cat 3) | 0.330 | .620 | .898 | .150 |
| Seek shaded areas | Maternal Age | 0.010 | .735 | 0.031 | .305 |
|  | Education (cat 2) | 0.828 | .133 | 1.220 | **.006** |
|  | Education (cat 3) | 0.559 | .379 | 1.678 | .**006** |
| Cooler building | Maternal Age | -0.035 | .442 | -0.020 | .411 |
|  | Education (cat 2) | -0.203 | .732 | 0.263 | .492 |
|  | Education (cat 3) | -0.253 | .706 | 0.287 | .530 |
| Used Mcako* | Maternal Age | na | na | na | na |
|  | Education (cat 2) | na | na | na | na |
|  | Education (cat 3) | na | na | na | na |
| Worked less | Maternal Age | 0.022 | .515 | 0.080 | **.010** |
|  | Education (cat 2) | -0.584 | .269 | 0.213 | .594 |
|  | Education (cat 3) | -0.716 | .236 | 0.320 | .539 |
| Bathed more | Maternal Age | -0.006 | .845 | 0.018 | .604 |
|  | Education (cat 2) | 0.254 | .451 | -0.200 | .655 |
|  | Education (cat 3) | -0.381 | .569 | -0.102 | .865 |
| Used fan | Maternal Age | -0.009 | .755 | 0.048 | .092 |
|  | Education (cat 2) | 0.269 | .592 | .563 | .208 |
|  | Education (cat 3) | 0.974 | .069 | 1.239 | **.022** |
| Used air con | Maternal Age | na | na | -0.024 | .586 |
|  | Education (cat 2) | na | na | 0.526 | .621 |
|  | Education (cat 3) | na | na | 2.069 | .059 |
| Wore light and loose clothing | Maternal Age | 0.043 | .173 | 0.013 | .734 |
|  | Education (cat 2) | 0.821 | .134 | 1.235 | **.008** |
|  | Education (cat 3) | 0.655 | .280 | 1.390 | .057 |
| Opening windows | Maternal Age | 0.038 | .083 | 0.035 | .330 |
|  | Education (cat 2) | 0.229 | .595 | 0.675 | .180 |
|  | Education (cat 3) | 0.477 | .304 | 1.019 | .133 |
| Cold water immersion | Maternal Age | 0.017 | .674 | 0.063 | **.019** |
|  | Education (cat 2) | 0.162 | .840 | 0.270 | .542 |
|  | Education (cat 3) | 0.278 | .749 | 0.022 | .968 |
| Wear a hat | Maternal Age | 0.018 | .529 | 0.068 | **.017** |
|  | Education (cat 2) | -0.305 | .477 | 1.387 | **<.001** |
|  | Education (cat 3) | -0.699 | .118 | 1.364 | **.017** |
| Use umbrella | Maternal Age | -0.031 | .406 | -0.005 | .916 |
|  | Education (cat 2) | 0.892 | .130 | 0.792 | .180 |
|  | Education (cat 3) | 0.360 | .581 | 1.590 | .078 |
| Stay indoors | Maternal Age | 0.006 | .837 | 0.007 | .740 |
|  | Education (cat 2) | 0.294 | .498 | 0.591 | .111 |
|  | Education (cat 3) | 0.077 | .896 | 1.431 | **.011** |
| Do nothing | Maternal Age | na | na | na | na |
|  | Education (cat 2) | na | na | na | na |
|  | Education (cat 3) | na | na | na | na |

***Table 5***. Postpartum cohort generalised estimating equation effect estimates assessing how mothers kept their **babies cool** during hot days comparing **midline to baseline** results. Adjusted for covariates: maternal age and education. Na represents model having insufficient data to execute the analysis.

*Mcako is type of clay high in minerals that is used as skin protection or often also ingested

| Behaviour | n baseline | n midline | p | OR | 95% CI |
| --- | --- | --- | --- | --- | --- |
| Breastfed more frequently | 57/101 | 63/83 | .316 | 1.28 | 0.79 - 2.09 |
| Gave baby more water | 55/101 | 59/83 | .449 | 1.81 | 0.77 - 1.82 |
| Gave baby more fruits | 17/101 | 21/83 | .345 | 1.31 | 0.75 - 2.27 |
| Used Mcako*/clay/ibovu | 0/101 | 2/83 | na | na | na |
| Sought shaded/cool areas | 43/101 | 59/83 | **.020** | 2.02 | 1.12 - 3.65 |
| Bathed baby more frequently | 68/101 | 67/83 | .873 | 0.96 | 0.55 - 1.66 |
| Used fan | 17/101 | 18/83 | .818 | 1.08 | 0.58 - 2.01 |
| Used air conditioning | 3/101 | 3/83 | na | na | na |
| Dressed baby in light & loose clothing | 91/101 | 76/83 | **.002** | 0.33 | 0.16 - 0.66 |
| Opened windows | 86/101 | 73/83 | **.024** | 0.88 | 0.79 - 0.98 |
| Kept baby indoors | 73/101 | 57/83 | .**020** | 0.49 | 0.27 - 0.89 |
| Did nothing | 0/101 | 0/83 | na | na | na |
| Other | 2/101 | 22/83 | **<.001** | 13.93 | 3.06 - 63.37 |

***Table 6****. Overall group covariate* *Wald χ² statistic with associated significance value assessing the question: Thinking about the last day when it was* ***hot what did you do to protect your baby from the heat****?. Education covariate was adjusted due to too few observations in each category to the following: 1 = some primary school, completed primary school, some high / secondary school, 2 = completed high / secondary school, 3 = some tertiary / university / college education, completed tertiary / university / college education. Highlighted in bold are the results that are significant on an overall group covariate level. Na indicates when model has too few observations to run the analysis.*

|  |  | | | **Endline** | | | **Midline** | |
| --- | --- | --- | --- | --- | --- | --- | --- | --- |
| **Heat Symptom** | **Covariate** | | | **Beta** | | **p** | **Beta** | **p** |
|  |  | **postpartum** | | | | | **postpartum** |  |
| Breastfeeding | Maternal Age | | -0.008 | | .783 | | 0.003 | .909 |
|  | Education (cat 2) | | 0.024 | | .953 | | 0.089 | .810 |
|  | Education (cat 3) | | -0.582 | | .292 | | 0.698 | .202 |
| Give baby more fluids | Maternal Age | | -0.030 | | .312 | | 0.022 | .452 |
|  | Education (cat 2) | | -0.621 | | .145 | | 0.921 | .056 |
|  | Education (cat 3) | | -0.804 | | .094 | | 0.375 | .497 |
| Give baby more fruits | Maternal Age | | -0.056 | | .117 | | 0.052 | .159 |
|  | Education (cat 2) | | -1.040 | | .135 | | 0.992 | .180 |
|  | Education (cat 3) | | 1.663 | | **.030** | | 1.110 | .148 |
| Used Mcako | Maternal Age | | na | | na | | na | na |
|  | Education (cat 2) | | na | | na | | na | na |
|  | Education (cat 3) | | na | | na | | na | na |
| Shade baby | Maternal Age | | -0.022 | | .426 | | 0.049 | .053 |
|  | Education (cat 2) | | -0.941 | | **.043** | | 1.589 | **<.001** |
|  | Education (cat 3) | | -1.525 | | **.006** | | 1.899 | **<.001** |
| Bathe baby | Maternal Age | | -0.037 | | .151 | | 0.015 | .598 |
|  | Education (cat 2) | | -0.330 | | .434 | | 0.056 | .890 |
|  | Education (cat 3) | | -0.991 | | **.049** | | 0.281 | .580 |
| Used fan | Maternal Age | | -0.054 | | .081 | | 0.085 | **.026** |
|  | Education (cat 2) | | -0.456 | | .363 | | 0.738 | .201 |
|  | Education (cat 3) | | -1.508 | | **.007** | | 1.482 | **.024** |
| Used air conditioning | Maternal Age | | na | | na | | na | na |
|  | Education (cat 2) | | na | | na | | na | na |
|  | Education (cat 3) | | na | | na | | na | na |
| Clothing baby | Maternal Age | | -0.022 | | .542 | | 0.025 | .502 |
|  | Education (cat 2) | | -0.320 | | .542 | | 0.492 | .339 |
|  | Education (cat 3) | | -1.397 | | .062 | | 1.071 | .187 |
| Opening windows | Maternal Age | | 0.004 | | .490 | | 0.005 | .289 |
|  | Education (cat 2) | | 0.083 | | .242 | | 0.092 | .178 |
|  | Education (cat 3) | | 0.220 | | .**002** | | 0.169 | **.044** |
| Kept baby indoors | Maternal Age | | -0.006 | | .814 | | 0.018 | .440 |
|  | Education (cat 2) | | -0.509 | | .203 | | 0.561 | .120 |
|  | Education (cat 3) | | -1.491 | | **.004** | | 1.141 | .023 |
| Did nothing | Maternal Age | | na | | na | | na | na |
|  | Education (cat 2) | | na | | na | | na | na |
|  | Education (cat 3) | | na | | na | | na | na |
| Other | Maternal Age | | -0.017 | | .619 | | -0.021 | .577 |
|  | Education (cat 2) | | -0.393 | | .615 | | -0.123 | .836 |
|  | Education (cat 3) | | -0.491 | | .595 | | -0.674 | .405 |

***Table 7****.* Self-reported behaviour changes due to EWS reported by pregnant and postpartum cohort.

| Self-reported behaviour changes due to EWS alerts | | | | |
| --- | --- | --- | --- | --- |
|  | Pregnant | | Postpartum | |
|  | n | Percent | n | Percent |
| No – slight change | 2 | 2.8 | 4 | 4.9 |
| Moderate change | 21 | 30.0 | 9 | 11.0 |
| Large – very large change | 47 | 67.2 | 68 | 82.9 |
| Do not know | 0 | 0 | 1 | 1.2 |
| Total | 70 | 100 | 82 | 100 |

*Fig 1*: Knowledge increase on heat effects on pregnancy health

*Fig 2*: Knowledge increase on heat effects on infant health

*Fig 3: PSSUQ summed overall score, with a lower score indicating a more positive outcome.*

*Fig 4: PSSUQ system usefulness, with a lower score indicating a more positive outcome.*

*Fig 5: PSSUQ Information Quality, with a lower score indicating a more positive outcome.*

*Fig 6: PSSUQ interface quality, with a lower score indicating a more positive outcome.*

**Appendix**

**7-in app EWS questions:**

1. Apart from completing this survey, did you access the MotherHeat Alert app in the past 7 days?

Yes, No

2. On a scale of 1 to 5, please rate how easy you have found it to use the app.

1 (Very easy), 2 (Easy), 3 (Neutral), 4 (Difficult), 5 (Very difficult)

3. Was the message you received relevant for your situation?

Yes, No

4. Did you follow the advice provided?

Yes, No

5. Thinking about the weather, how do you feel right now?

3 Hot, 2 Warm, 1 Slightly warm, 0 Neutral, -1 Slightly cool, -2 Cool, -3 Cold

6. Please rate how comfortable the weather feels for you right now?

0 Comfortable, 1 Slightly uncomfortable, 2 Uncomfortable, 3 Very uncomfortable, 4 Extremely uncomfortable

7. Please rate your current level of wetness due to sweating.

1 (Dry), 2 (A little wet), 3 (Wet), 4 (Extremely wet)

**Post-Study System Usability Questionnaire (PSSUQ)**

8. Overall, I am satisfied with how easy it is to use this system.
9. It was simple to use this system.
10. I was able to complete the tasks and scenarios quickly using this system.
11. I felt comfortable using this system.
12. It was easy to learn to use this system.
13. I believe I could become productive quickly using this system.
14. The system gave error messages that clearly told me how to fix problems.
15. Whenever I made a mistake using the system, I could recover easily and quickly.
16. The information (such as online help, on-screen messages, and other documentation) provided with this system was clear.
17. It was easy to find the information I needed.
18. The information was effective in helping me complete the tasks and scenarios.
19. The organization of information on the system screens was clear
20. The interface of this system was pleasant.
21. I liked using the interface of this system.
22. This system has all the functions and capabilities I expect it to have.
23. Overall, I am satisfied with this system.

Ranging from strongly agree (1) to strongly disagree (7)

1 to 16: Overall Questions

1 to 6: System Usefulness (SYSUSE)

Questions 7 to 12: Information Quality (INFOQUAL)

Questions 13 to 16: Interface Quality (INTERQUAL)

<https://uiuxtrend.com/wp-content/uploads/PSSUQ-Questionnaire-PDF-Template.pdf>
